# Aging-Related Changes in the Injury Response of the Peripheral Nervous System

**DOI:** 10.1007/s12264-025-01564-4

**Published:** 2026-02-24

**Authors:** Yue-Yan Cen, Mu-Yun Wang, Qin-Xuan Song, Xin-Lin Gao, Cheng Zhou, Chun-Jie Li, Fei Liu, Yan-Yan Zhang, Jie-Fei Shen

**Affiliations:** 1 State Key Laboratory of Oral Diseases, National Clinical Research Center for Oral Diseases, National Center for Stomatology, West China School of Stomatology, Sichuan University, Chengdu, 610041 China; 2 Department of Prosthodontics, West China Hospital of Stomatology, Sichuan University, Chengdu, 610041 China; 3 Laboratory of Anesthesia and Critical Care Medicine, Translational Neuroscience Center, West China Hospital of Sichuan University, Chengdu, China; 4 Department of Head and Neck Oncology, West China Hospital of Stomatology, Sichuan University, Chengdu, China

**Keywords:** Aging, Injury, Neural regeneration, Mitochondria, Axonal transport

## Abstract

Peripheral nerve injury (PNI) significantly impairs patients’ quality of life, with elderly individuals experiencing particularly severe consequences due to aging-related declines in neuronal injury response and repair capabilities. Processes of the generation and transmission of injury signals, axonal disruption, initiation of regeneration, and the elongation of regenerating axons, as well as the subsequent reinnervation by these axons, are all significantly influenced by aging. These alterations are closely associated with changes in mitochondrial function, neuronal transport systems, a persistent inflammatory milieu, and various microenvironmental non-neuronal cells. Therefore, this review synthesized the pivotal role of aging in the multifaceted regulation of the nervous system following PNI and highlighted promising molecular regulatory mechanisms in the signaling pathways. Furthermore, it identified critical areas for future research, including unresolved questions in age-associated injury responses, potential targets for pharmacological intervention, and emerging therapeutic strategies meriting consideration for research and development.

## Introduction

The peripheral nervous system (PNS) encompasses cranial, visceral, and spinal nerves, along with their associated ganglia. Peripheral nerve injury (PNI) is often categorized as neurapraxia, axonotmesis, and neurotmesis [1]. Research on neural injury responses has predominantly examined structural damage to neurons [2–6]. PNI has a high prevalence and arises from trauma (25%–76%) [7, 8], medication-induced injury (60%–85%), and various diseases such as diabetes (≥50%) and cancer (70–100%) [9], etc. [10–13]. However, current therapeutic approaches remain inadequate in fully restoring the damaged axons, leading to various motor and sensory deficits, which may ultimately result in permanent disability [14–16].

Aging is characterized by a gradual decline in the functionality of tissues as well as organs and a decrease in homeostasis, involving extremely complex physiological and pathological mechanisms. Numerous studies have shown that the speed and degree of degeneration and regeneration following PNI are negatively correlated with age, with observable changes at both morphological and molecular levels [17–22]. This phenomenon is caused by both aging neurons and the associated senescent microenvironment, which includes various cell types [23]. Its precise mechanisms continue to be explored.

There are ten hallmarks of aging in the nervous system that modulate the responses of PNI to varying degrees [24]. Among these, dysregulation of neuronal calcium homeostasis, impaired molecular waste disposal, impaired adaptive stress response signaling, and impaired DNA repair are all important factors in the decreased responsiveness of PNI [4]. In addition, the process of PNI response is also influenced by several other age-related changes, including mitochondrial dysfunction and its resulting dysregulation of oxidative damage and energy metabolism, abnormalities in cytoskeletal structure and function, and the hyperinflammatory state [5]. Non-neuronal elements, particularly Schwann cells (SCs) and macrophages, also contribute to the age-related modulation of the PNI response. However, these changes have not been systematically elucidated. This review focuses on the specific manifestations of these hallmark aging changes in PNI models and how they affect the cellular and molecular mechanisms involved in neuronal response.

## Literature Search Strategy

This review was conducted through a comprehensive search of the literature available in PubMed, Web of Science, and Google Scholar databases up to August 2025. The search terms included combinations of the following keywords: aging, peripheral nerve injury, axon regeneration, calcium signaling, autophagy, Wallerian Degeneration, mitochondrial dysfunction, axonal transport, Schwann cells, macrophages, neuroinflammation, and neuromuscular junction. Articles were selected based on their relevance to aging-related changes in the peripheral nervous system response to injury, with a focus on molecular mechanisms, cellular interactions, and potential therapeutic interventions. Both original research and review articles were considered, with priority given to studies published in the past decade, although seminal earlier works were also included for historical context. Non-English publications and studies not directly related to aging or peripheral nerve injury were excluded.

## Aging Affected Degenerative Responses for PNI

Following PNI, neurons experience retrograde degeneration at the proximal site and Wallerian Degeneration of the distal axons, which are also necessary prerequisites for nerve repair [18, 25–29]. Early dysregulation of Ca^2+^ responses and impaired autophagic clearance play crucial roles in neurodegeneration. They participate upstream in response mechanisms, inhibit neuro-inflammation, and provide the necessary material and spatial conditions for nerve regeneration [19]. Moreover, it was found that aging significantly impacted these critical neuronal injury responses.

### Dysregulation of the Ca^2+^ Injury Response

Following an axonal injury, the axonal membrane ruptures. This breach promptly activates diverse calcium channels, resulting in a swift rise in intracellular Ca^2+^ concentrations [30–33]. Numerous evidence suggest that the subthreshold or resting state Ca^2+^ spikes occurring post-injury play an important role in the processes of degeneration and subsequent regeneration [27, 34–37]. The immediate and well-regulated influx of Ca^2+^ following axotomy is a critical trigger for the orderly progression of degeneration. This view is encapsulated in the "calcium hypothesis" of neurodegeneration [38]. Localized calcium overload triggers a widespread neuronal response characterized by mitochondrial dysfunction [39], activation of calcium-activated chloride currents [40], cleavage of sodium channels [41], mediation of DNA demethylation [6, 38, 42], modulation of axonal transportation [43], initiation of Wallerian Degeneration [5, 6, 26, 27, 42, 44], promotion of growth cone formation [45–48], and enhancement of axonal regeneration [32, 49–51].

**Fig. 1:**
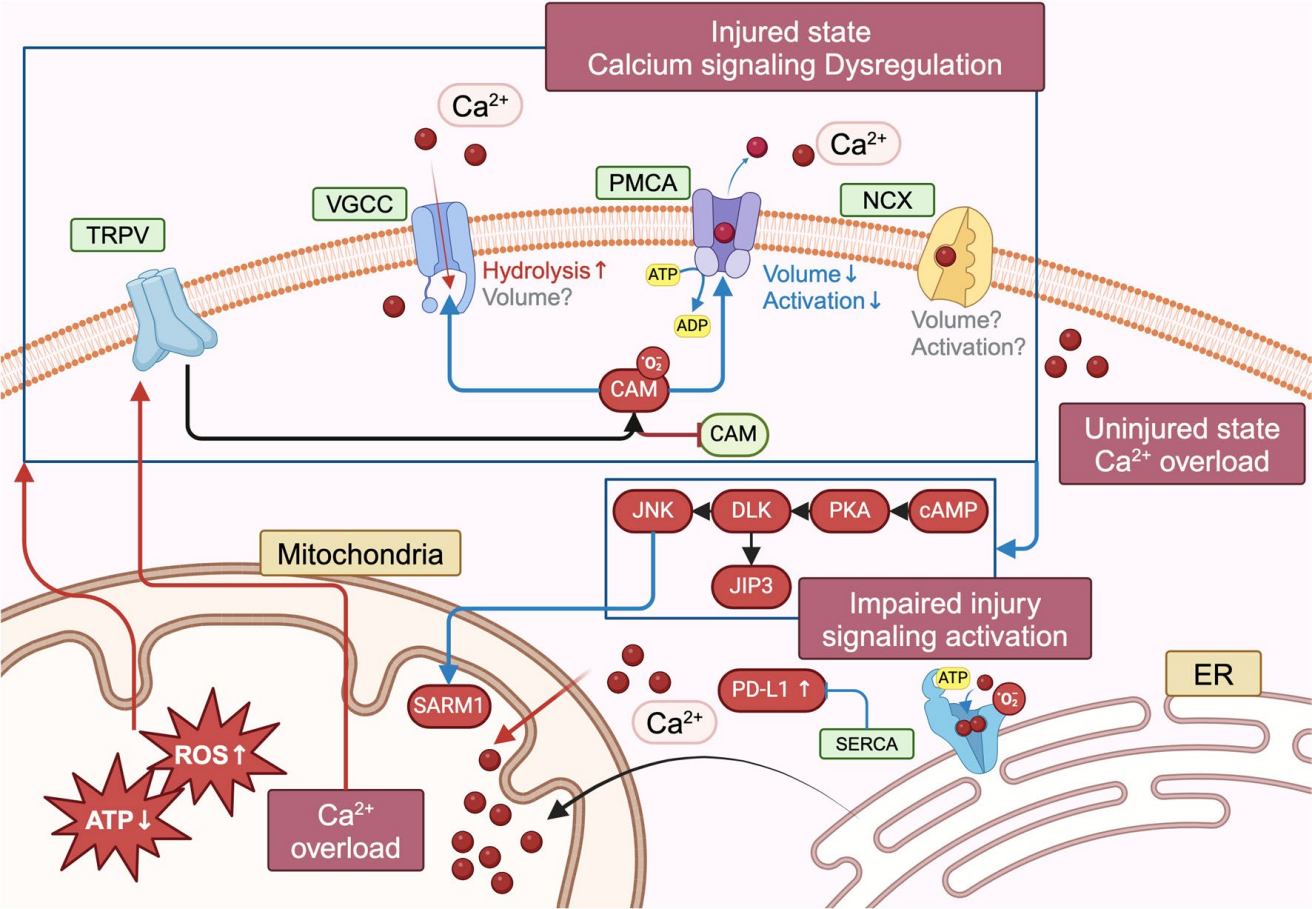
Aging-related disturbances in calcium homeostasis and the diminution of calcium ion regulatory pathways after injury. Aging produces multiple effects on calcium channels, including those in neuronal axon membranes and organelle membranes, leading to calcium overload in the cytoplasm and inside organelles represented by mitochondria. At the same time, the regulation of calcium signaling, a typical early response after injury, is significantly altered. Thus, aging significantly affects a range of calcium-associated physiological or injury response functions. The arrows in the figure demonstrate regulatory processes that are up-regulated (red) or down-regulated (blue) by the aging state.

In peripheral neurons, a key feature of aging is the disruption of calcium homeostasis and the improper regulation of calcium levels [35, 52, 53] (Fig. 1). This disruption is manifested by elevated resting cytoplasmic Ca^2+^ concentrations, increased Ca^2+^ release from the endoplasmic reticulum (ER), and enhanced Ca^2+^ translocation from the ER to the mitochondria [54]. Additionally, there is a noticeable alteration in the expression patterns of proteins that are pivotal to maintaining calcium balance [55]. Critically, injury exacerbates this age-related calcium dysregulation—disrupting the regulatory mechanisms of calcium-signaling proteins and channel activities that mediate post-injury responses—and drives excessive, sustained Ca^2^⁺ elevations [56–58]. This transformation shifts calcium from a constructive trigger (e.g., for orderly Wallerian degeneration progression) to a destructive force that impairs injury repair.

Voltage-gated calcium channels (VGCCs), particularly L-type channels, are integral to the axonal membrane and open upon membrane depolarization to mediate Ca^2+^ influx from the extracellular space [59]. They are essential for regulating the growth cone cytoskeleton and stimulating sustained axonal growth after neuronal injury [46, 47]. In aging, the expression of L-type VGCC is upregulated [60, 61], leading to an exaggerated and dysregulated Ca^2+^ influx upon axolemma rupture. This age-related hyperactivation triggers an initial toxic surge of Ca^2+^ that overwhelms the neuron's homeostatic capacity. The current findings conclude that this upregulation may be related to abnormal firing, an aging-related increase in intracellular reactive oxygen species (ROS), and a dramatic increase in intermediate channel proteolysis [60, 62–64], resulting in an exaggerated Ca^2+^ influx upon membrane rupture. This age-dependent hyperactivation exceeds the buffering capacity of aged neurons. However, the characterization of aging-related changes in VGCC protein levels remains to be fully elucidated, probably due to technical and methodological challenges in quantitative studies that utilize specific antibodies targeting VGCC [53].

Beyond the increased influx of Ca^2+^, aging-related failure to clear cytosolic Ca^2+^ represents another detrimental factor. The plasma membrane Ca^2+^-ATPase (PMCA) pump is a high-affinity, low-capacity transporter that uses adenosine triphosphate (ATP) hydrolysis to actively extrude Ca^2+^ against its concentration gradient, fine-tuning cytosolic Ca^2+^ levels under resting and low-load conditions [65]. It has been well documented that the activity and quantity of PMCA, especially PMCA2 and PMCA3, the so-called "fast" isozymes, decline with age, leading to increased intracellular calcium levels and diminished regulatory capacity [52, 66]. Calmodulin (CaM), a key activator of PMCA, undergoes extensive oxidative modification in aging organisms. This modification not only greatly reduces the biological activity of PMCA but also prevents unoxidized CaM from activating PMCA, thereby indirectly leading to a decline in PMCA's calcium-regulatory function [67–69]. This failure constitutes a primary defect in clearing the injury-induced Ca^2+^ load, allowing concentrations to remain pathologically elevated. Additionally, PMCA has been implicated in post-traumatic neuropathic pain (NP) [70]. Further research is needed to determine whether there is a direct correlation between the age-induced reduction in PMCA activity and the development of NP following injury.

Residing on the ER membrane, the sarcoplasmic/endoplasmic reticulum Ca^2^⁺ ATPase (SERCA) pump re-accumulates cytosolic Ca^2^⁺ into the ER stores, maintaining a high intraluminal Ca^2^⁺ concentration crucial for signaling and protein processing. It also acts as a significant buffer during Ca^2^⁺ overload. Measurements in aging models reveal a substantial loss of SERCA activity [71], due largely to oxidative modification of its cysteine residues [72–74]. This impairment disrupts ER Ca^2^⁺ homeostasis and eliminates a major intracellular sequestration route, further contributing to sustained cytosolic Ca^2^⁺ elevation. Furthermore, diminished SERCA activity has been correlated with the upregulation of programmed cell death receptor PD-L1 [75], which may contribute to the increased vulnerability of aging peripheral neurons to injury.

Mitochondrial calcium channels and the ER-mitochondrial transfer function of Ca^2^⁺ are also recognized as aging-influenced factors. They have been suggested as potential therapeutic targets for central nervous system (CNS) lesions [37, 76]. These channels receive calcium signaling regulation and, at the same time feedback on the signal, playing a crucial role in signal amplification [77–80]. For instance, the overloading of Ca^2^⁺ in mitochondria leads to their dysfunction and stimulates signaling pathways represented by transient receptor potential vanilloid (TRPV) activation [81, 82]. TRPV channels, in turn, modulate cellular activity through signals like CaM [37, 83]. Besides, in young neurons, mitochondrial calcium uptake helps buffer cytosolic loads and can support energy production for repair processes [84]. In aging, however, mitochondrial calcium overload, due to impaired regulation, directly precipitates mitochondrial permeability transition pore (mPTP) opening and ROS overproduction [85, 86]. This age-specific vulnerability turns mitochondria from buffers into amplifiers of the injury signal, releasing additional pro-degenerative factors [87].

In studies across various biological systems, it has been found that the volume and function of other calcium-regulated channels are also associated with age [88, 89]. The sodium-calcium exchanger (NCX) operates as a low-affinity, high-capacity secondary transporter that utilizes the Na⁺ electrochemical gradient to extrude one Ca^2^⁺ ion for the import of three Na⁺ ions [90]. It is particularly critical for relieving large Ca^2^⁺ loads, such as those following injury [91]. Although the specific alterations of NCX in aging PNI have not been fully elucidated, its functional impairment can act synergistically with PMCA and SERCA deficiencies, severely compromising the cell's capacity to recover from massive Ca^2^⁺ influx [92, 93]. In-depth investigation of NCX-mediated dysregulation of calcium homeostasis holds significant promise for unveiling novel therapeutic targets for peripheral nerve injury in elderly patients [94, 95].

Aging is associated not only with changes in the volume and activity of calcium channels but also with a reduction in the intraneuronal expression of calcium-binding proteins and their associated signaling pathways. This downregulation further intensifies the dysregulation of neuronal calcium levels during aging [33, 96, 97]. Therefore, aging does not merely modulate the calcium injury signal; it fundamentally corrupts it. The combined effect of increased influx and profoundly impaired efflux/sequestration in aged neurons creates a perfect storm of calcium dysregulation that excessively amplifies the degenerative cascade, leading to more rapid and complete axonal disintegration compared to young neurons [98]. It has also been observed that PNI induces calcium fluctuations in glial cells, which in turn influence neuronal regeneration. However, the exact molecular mechanisms involved in the bidirectional signaling exchanges between the glia and neurons and the exact molecular mechanisms have not yet been clarified [34]. Furthermore, a growing body of research has identified the aging-associated calcium-dynamic imbalance as one of the major factors triggering diseases in the CNS [99, 100]. The relationship between PNS disorders and aging-related decreases in calcium regulatory activity after injury is a critical area that merits future investigation.

### Dysregulation of Neuronal Autophagy

Autophagy plays a crucial role as a cellular mechanism that facilitates the degradation of dysfunctional organelles and protein aggregates in neurons after PNI. Additionally, it is involved in the signaling pathways that regulate the axonal injury response [28, 101]. Insufficient or failed autophagic response after PNI impedes regeneration [102, 103]. Aging imposes a basal suppression of autophagy and, crucially [104], blunts its inducibility upon injury [105], creating a failure in this critical clearance mechanism. Research has demonstrated that axonal damage in aging neurons does not trigger the formation of autophagic vesicles, suggesting that neurons in an aged state lose their capacity to activate autophagy in response to injury [19, 106–108].

**Fig. 2:**
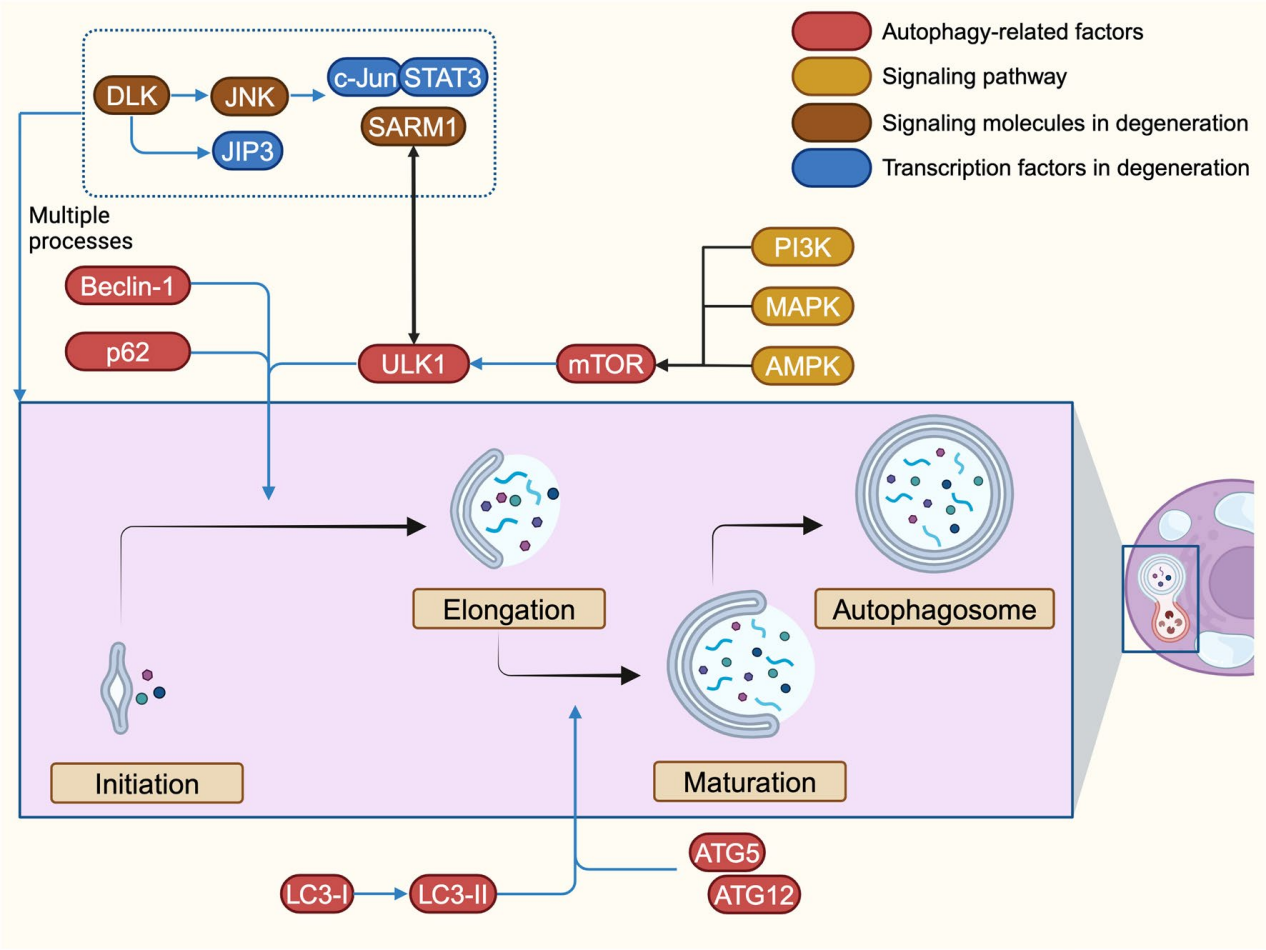
Aging-related autophagy signaling pathways both in physiological and injured states. Classical autophagy pathways of peripheral neurons, including those in the physiological state and autophagy-related signaling pathways that are specifically upregulated in response to injury, are widely affected by the aging process, with the overall manifestation of autophagy inhibition and dysregulation. Blue arrows represent regulatory pathways that have been shown to be downregulated by aging.

The Phosphatidylinositol 3-kinase (PI3K)/Mitogen-activated protein kinase (MAPK)/AMP-activated protein kinase (AMPK) - mammalian target of rapamycin (mTOR) - UNC-51-like kinases 1 (ULK1) signaling axis plays a pivotal role in the regulation of autophagy [101, 109, 110] (Fig. 2). PI3K, MAPK, AMPK, and mTOR are intimately involved in the bidirectional regulation of neural aging [111, 112], and their activities all decrease with age [113–115]. In studies of skeletal muscle and plasma, it has also been reported that aging is linked to a decrease in ULK1 activation, which leads to a reduction in autophagic flux [101, 116].

In the model of PNI, the impact of aging on neuronal autophagy is modulated by multiple regulatory mechanisms. The dual leucine zipper-bearing kinase (DLK) was found to be necessary and sufficient for the initiation of autophagy activation after injury [19]. DLK activation is dependent on the cyclic adenosine monophosphate (cAMP) - protein kinase A (PKA) pathway, which is activated by axonal injury-responsive calcium waves [4]. The efficacy of the signaling pathway is observed to diminish with aging [117], potentially leading to a suppression of neuronal autophagy. In aged organisms, overexpression of DLK has been demonstrated to counteract the age-related reduction in autophagy activation and to foster axonal regeneration [19]. Downstream proteins of DLK, including c-Jun N-terminal kinase (JNK) and JNK-interacting protein 3 (JIP3) [4, 104], are also pivotal in facilitating autophagy [118]. Similarly, their expressions are reduced in aging nerves [119]. Furthermore, the expression of JNK and its downstream signals involved in the autophagy process, such as sterile alpha and toll-interleukin receptor motif-containing 1 (SARM1) [101], transcription 3 (STAT3) [120], and c-Jun [121], is affected by aging, thereby dampening the autophagic response to PNI through various mechanisms. Other autophagy-related proteins, including Beclin-1, p62, microtubule-associated protein 1 light chain 3 (LC3)-II/LC3-I ratio, and autophagy-related gene (ATG)5 and ATG12, were also shown to be downregulated in aging neurons [122] (Fig. 2), but the key defect may lie in signaling upstream of autophagy initiation.

In particular, the protein SARM1, known for its prodegenerative properties, plays a pivotal role in neuronal response subsequent to injury [123]. It is intricately involved in various signaling pathways that govern a spectrum of cellular responses, including calcium signaling regulation, axonal degeneration and regeneration, as well as inflammatory homeostasis [123–125]. The interaction between ULK1 and SARM is particularly significant, as it forms a key link in the involvement of autophagy in mediating axon degeneration [101, 124, 126] (Fig. 2). Studies have shown that inhibition of autophagy attenuated SARM1 accumulation in injured nerves [101], implying that aging factors may regulate autophagy through PI3K/MAPK/AMPK-mTOR-ULK1 signaling, thereby influencing neuronal response to PNI through the SARM1 pathway and other interconnected processes [3]. Considering the comprehensive role of SARM1, subsequent research may explore whether it serves as a viable therapeutic target for ameliorating aging-associated alterations in PNI [123].

Injury response signaling molecules affected by aging are widely present in the regulatory pathways of autophagy, which has a complex, multilevel effect on PNI responses. This age-dependent failure to activate autophagy has two major consequences: inefficient clearance of damaged components and impaired provision of resources [127, 128]. Autophagy could be an important therapeutic target for the rehabilitation of elderly patients after PNI.

### Dysregulation of Wallerian Degeneration

Wallerian Degeneration is a critical process following axonal injury in the PNS. This process is characterized by the disintegration of severed axons, degradation of myelin sheaths, and the subsequent clearance of myelin debris facilitated by SCs and macrophages, ultimately promoting the regeneration of axons [29]. Wallerian Degeneration acts as a protective mechanism of programmed axon degeneration, the complex molecular mechanisms of which are still under investigation [2]. Furthermore, this significant response to PNI is also severely affected by aging. In injury models of aging nerves, there is a notable reduction in the levels of cytokines, chemokines, as well as the number of non-myelinating SCs and macrophages [129, 130]. These changes lead to suppression of Wallerian Degeneration.

During Wallerian Degeneration, SCs in distal nerve segments undergo division and proliferation, reprogramming to revert to a non-myelinating phenotype that facilitates repair processes. Non-myelin SCs secrete large amounts of growth-associated factors, recruit macrophages, and remove myelin debris to ensure unobstructed space for axon growth and extension [129, 131, 132]. Additionally, they form cellular bridges at sites of axonal discontinuity, known as the Büngner band, which facilitate the guidance of subsequent axonal regeneration [133]. These findings highlight the pivotal role of SCs in the responses associated with Wallerian Degeneration in PNI.

In summary, Wallerian Degeneration is not merely a passive disintegration but an active process orchestrated by SCs and macrophages. Aging severely disrupts this orchestration, primarily by impairing the phenotypic plasticity of SCs and their cross-talk with immune cells, within a backdrop of a hostile microenvironment [134]. In vitro experiments have demonstrated that aging SCs exert a pronounced inhibitory influence on axonal regeneration [134–136]. The failure of myelinating SCs to reprogram effectively is a critical early defect in aged nerves. Molecular assays of the sciatic nerve have revealed that aging SCs display a broad impairment of dedifferentiation, myelin clearance, and macrophage recruitment [131], along with transcriptional dysregulation of genes pivotal to nerve regeneration [137]. Recent studies have pointed out that aging myelinating SCs exhibit reduced c-Jun expression after injury [134], resulting in an attenuated dedifferentiation capacity [138]. Moreover, the progression of myelinating SCs to an aging phenotype may correlate with the upregulation of p16, a factor that suppresses c-Jun function [134]. This process could be reversed pharmacologically or genetically, for instance, by augmenting c-Jun levels to ameliorate the functional deficits in aging myelinating SCs [134, 136]. Beyond the well-characterized c-Jun regulatory pathway, further research is warranted to elucidate other factors that govern the dedifferentiation potential of aging myelinating SCs and their post-differentiation functions, such as the secretion of signaling molecules and other aging-related modifications [136, 139–141]. Non-myelinating SCs rely on the Seh1 gene to maintain genomic stability and perform auxiliary roles in immune regulation. However, aging leads to Seh1 functional defects, triggering apoptosis and neurofibrillary degeneration, thereby similarly impairing its efficacy and weakening its aforementioned regenerative support capacity [142].

Beyond the well-characterized signaling dysregulation, aged Schwann cells also exhibit a significant loss of metabolic adaptability, which further compromises their repair-supportive functions. For instance, dysregulation of N-methyl-D-aspartate (NMDA) receptor-mediated metabolic pathways weakens their metabolic plasticity [143]. Furthermore, altered expression of glucose transporters and disrupted mitochondrial pyruvate metabolism disrupts the energy homeostasis essential for supporting axonal regeneration [144–146]. These metabolic disturbances form a vicious cycle with inflammation and cellular senescence [147–149].

Besides, the immune response can interfere with the ability of SCs to dedifferentiate and reprogram into a repair state following injury [150]. Macrophages are pivotal immune cells in the context of PNI and the subsequent repair processes. Different cell phenotypes can exhibit two completely different efficacies, pro-inflammatory (M1 phenotype) and anti-inflammatory (M2 phenotype) [151]. In response to PNI, macrophages not only remove myelin debris and modulate the activity of SCs in Wallerian Degeneration, but also polarize M2 and promote axonal growth by releasing large amounts of axonal regeneration-associated factors [25, 152].

Previous experiments have shown that aging significantly affects macrophage recruitment and phenotypic differentiation, with a significant decrease in the proportion of M2 phenotypic cells in the aging setting and a concurrent increase in the M1 phenotypes, which is associated with excessive inflammation in aging neurons [153–155]. This is not only due to intrinsic deficits but also influenced by the aging extracellular matrix (ECM). An age-related increase in ECM stiffness and cross-linking can physically hinder the migration of SCs and macrophages to the injury site, further delaying debris clearance [156]. Moreover, the accumulation of senescent cells (SCs, fibroblasts) within the microenvironment creates a persistent inflammatory state through the secretion of the senescence-associated secretory phenotype (SASP) [157]. This altered microenvironment profoundly affects macrophage function. The diminished secretion of key recruitment signals by aged SCs, such as monocyte chemotactic protein-1 (MCP-1) [158] and interleukin (IL)−17B/IL-17RB signaling pathway [159], leads to delayed and insufficient macrophage infiltration. Furthermore, the SASP-rich, pro-inflammatory microenvironment biases recruited macrophages towards a pro-inflammatory (M1) over an anti-inflammatory, pro-regenerative (M2) phenotype [160]. Experimental evidence suggests that functional defects in aging macrophages can be completely rescued by transplantation into a younger microenvironment, indicating that the intrinsic capacity of these macrophages to respond to injury is not significantly compromised [150]. Besides, research into the aging-related decline in myelin debris phagocytosis within the CNS has revealed that aging can disrupt the retinoid X receptor pathway, leading to impaired phagocytic activity of myelin debris by phagocytes. This effect can be pharmacologically reversed, suggesting the potential of this pathway as a target for therapeutic intervention [161]. However, the specific role of the retinoid X receptor pathway in the PNS requires further exploration, as the aforementioned study pertains specifically to the CNS environment, including microglia.

The aging environment can indeed have a multifaceted impact on the healing process following PNI. In addition to the above factors, the aging environment with impaired vascular function (including dysfunction of endothelial cells, smooth muscle cells, etc.), the state of the neuro-endothelial canal, and other macrophage recruitment signals may also influence the role of macrophages in Wallerian Degeneration and axon regeneration [4, 162].

## Aging Affected Neuronal Regenerative Processes

Axonal regeneration following PNI is one of the pivotal aspects of injury responses. It is well-established that the ability of axons to regenerate decreases markedly with increasing cellular or biological age [163]. This age-related decline is closely associated with various phases of recovery and regulatory mechanisms post-injury. It not only reflects the expression of regenerative capabilities but also serves as an intuitive evaluative index of the overall neuronal response to injury [4]. The decline in axonal regenerative capacity in aging neurons is significantly influenced by mechanisms of programmed cellular aging and alterations in related molecular functions [4, 164]. Here, we explored several important regulatory mechanisms in aging-related axonal regeneration.

### Axonal Regeneration Signaling Molecules and Transcription Factors

The successful transition from axonal degeneration to regeneration requires the efficient retrograde transport of injury signals to the soma to initiate a pro-regenerative transcriptional program [165]. The effects of aging on this process are complex, influencing various transcription factors that are essential to its function.

#### The Initiating Signal: DLK as a Central Hub

As discussed earlier, DLK is a master upstream regulator situated at the crossroads of degeneration and regeneration. Besides, DLK is a central signaling molecule that influences axonal degeneration, regeneration, and the development of chronic pain subsequent to PNI [166]. Its deletion or downregulation directly blocks the transport of retrograde injury signals and the accumulation of pro-regenerative signals in the dorsal root ganglion (DRG) cells, thereby obstructing axonal regeneration altogether. Reversal of this change also exhibits upregulation of axonal regenerative function [4, 166–169]. The change in DLK function due to aging has been described previously. Thus, the age-related blunting of the DLK response represents a critical early failure that prevents the initiation of the entire regenerative cascade, effectively silencing the neuron's intrinsic repair program.

#### The Retrograde Signal Carriers: JNK, ERK, and STAT3

**Fig. 3:**
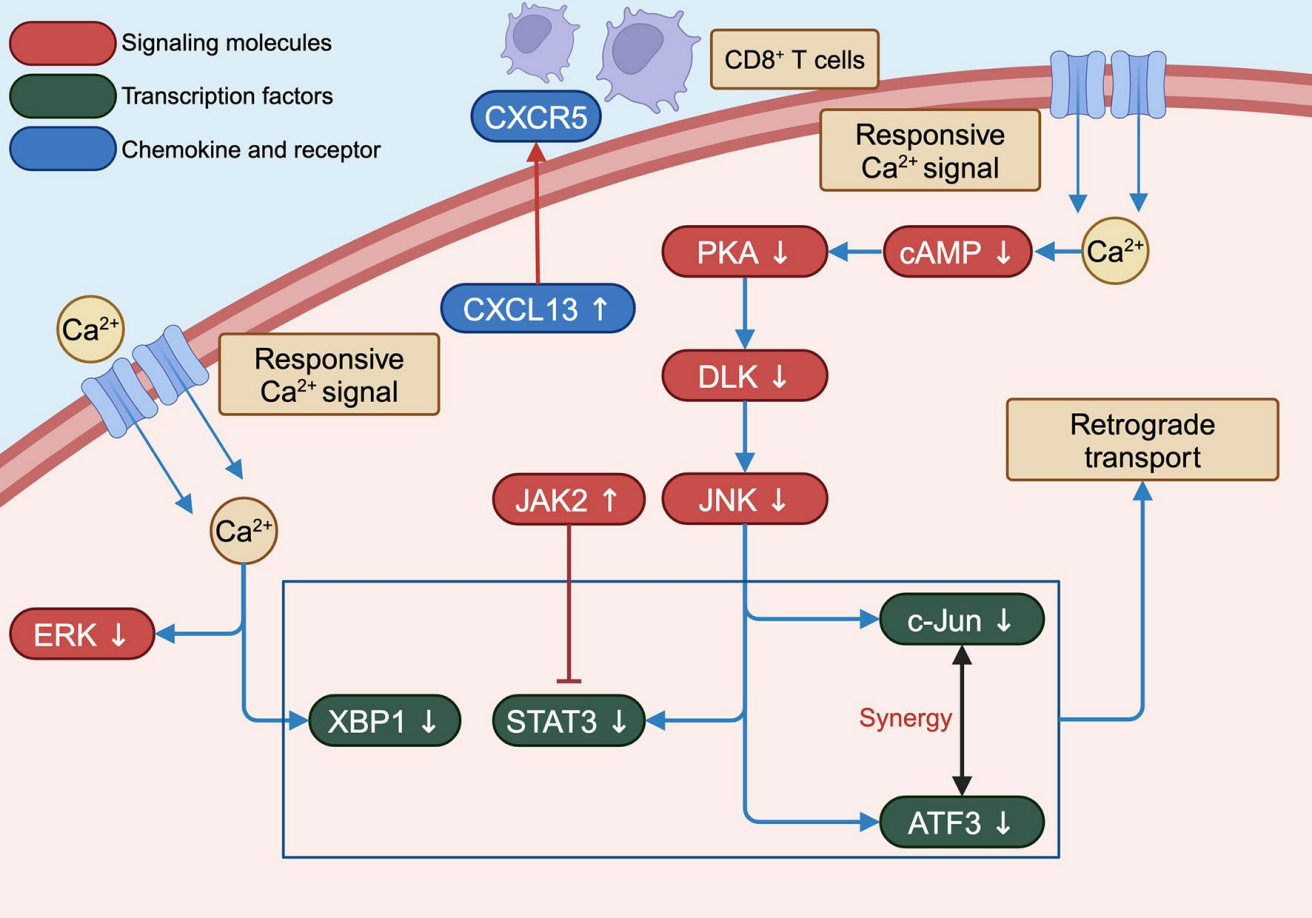
Molecules and factors participate in regeneration and their aging-related changes. Some of the signaling molecules and transcription factors that are highly relevant to axonal regeneration have been shown to be significantly affected by the aging state. The calcium signaling-activated cAMP-PKA-DLK signaling pathway is important in regulating the initiation of axon regeneration, and several transcription factors, including c-Jun, ATF3, and STAT3, are affected by its aging-related changes. XBP1 also exhibits aging-associated downregulation. These transcription factors are also affected by the decreased retrograde transport function. In addition, CXCL13-CXCR5 is an important chemokine pathway in the decline of regenerative function in aging neurons. The blue and red arrows represent down- and up-regulation of regulatory signals in the aging state, respectively. White arrows indicate changes in the activity or volume of molecules and factors.

The DLK-JNK axis functions as a retrograde signal carrier. After PNI, activated JNK serves as an upstream signal for a variety of regulatory pathways that promote axonal regeneration (Fig. 3). As previously discussed, it plays a key role in neuronal autophagy and the activation of myelinating SCs—particularly their dedifferentiation into a repair-promoting phenotype—by mediating Jun phosphorylation, which is essential for axonal regeneration after PNI [170]. This c-Jun-related process occurs only in the PNS and is not activated in the CNS [171]. Similarly, calcium influx after injury activates importin-β, enabling it to bind transcription factors such as extracellular regulated protein kinase (ERK) and facilitate their retrograde transport. This represents another key pathway for axon extension that is downregulated in aging [172–174]. Aging severely impairs this axonal transport system, leading to diminished nuclear translocation of these critical signals [119]. The deletion of DLK and the attenuation of the JIP3-JNK retrograde signaling pathway also affect the retrograde transport of STAT3 [5]. STAT3, another downstream signaling module of DLK, is transported to the nucleus [175] and contributes to neuronal regeneration through multiple regulatory pathways [158, 175–182]. The expression and function of STAT3 are affected by multifaceted aging-related changes in the PNS [181], with the balance shifting toward detrimental effects [5]. First, hyperactivation of upstream regulators like JAK2 in aging can suppress the pro-regenerative expression of STAT3 [183, 184] (Fig. 3), while chronic inflammatory signals in the aged microenvironment may promote its pro-inflammatory phosphorylation [185]. Second, the function of STAT3 is cell-type-specific. Its beneficial function in neuronal regeneration may decline with age, whereas its detrimental pro-inflammatory role in non-neuronal cells (e.g., immune cells, cardiomyocytes) may become predominant [177, 179]. Third, transient, acute activation of STAT3 is likely reparative, whereas chronic, sustained activation—a hallmark of aged and inflamed tissues promotes pathological inflammation and inhibits regeneration [186, 187]. This age-related shift in STAT3 function from a pro-regenerative to pro-inflammatory factor exemplifies how aging can corrupt key signaling nodes. Therefore, the bidirectional role of STAT3 is not a contradiction but a consequence of the aged microenvironment [180, 188]. Understanding these determinants is crucial for developing therapies that can selectively modulate beneficial aspects of STAT3 signaling. The bidirectional role of STAT3 in aging is a burgeoning field of research, with the potential to identify STAT3 as a regulatory target for reversing neuronal aging-related behaviors [189, 190]. Collectively, aging disrupts the faithful retrograde transport of critical regeneration signals such as JNK, ERK, and STAT3, severing the communication line between the injury site and the nucleus and leaving the pro-regenerative transcriptional program unactivated.

#### The Nuclear Executors: c-Jun, ATF3, and SMADs

Upon reaching the nucleus, retrograde signals activate a core regenerative transcriptional program. c-Jun and activating transcriptional factor 3 (ATF3) are pivotal players that synergistically promote the expression of regeneration-associated genes (RAGs) and are uniquely efficacious in the PNS [4, 170, 171]. Additionally, calcium release can lead to the splicing and nuclear translocation of X-box-binding protein 1 (XBP1), where it participates in the transcription of axonal regeneration signals—a process also reported to be altered in aging [191, 192]. Aging is associated with a diminished and delayed upregulation of both c-Jun and ATF3, as well as compromised XBP1 signaling [119, 189]. This failure to mount a robust transcriptional response means that even if the injury signal is perceived, the genetic machinery to execute regeneration remains idle. Furthermore, these factors interact with members of the Sma- and Mad-related protein (SMAD) family, which are involved in cytoskeletal dynamics; however, the age-specific alterations in SMAD signaling after PNI remain an open question [193, 194].

Crucially, many of these factors play dual and opposing roles in degeneration and regeneration (e.g., DLK, JNK). The functional outcome likely depends on the dynamics and context of their activation [163, 195]. Aging disrupts this delicate balance by dampening beneficial regenerative functions (e.g., the pro-regenerative role of DLK, the neuroprotective role of STAT3) while failing to resolve—or even exacerbating—destructive degenerative functions (e.g., chronic inflammation driven by STAT3) [195, 196]. The result is a signaling network biased towards failure, explaining the profound regenerative deficit in the aged PNS.

### The Effect of Chemokines in the Mediation of Regeneration

Chemokines play a pivotal role in modulating various physiological responses to PNI, including inflammation, axonal regeneration, and pain [197, 198]. During successful regeneration, chemokines form spatial and temporal gradients that critically guide the directional growth of regenerating axons and orchestrate immune cell recruitment to the injury site [199]. However, aging is associated with widespread changes in the chemokine landscape. The precision of this chemokine-guided navigation is profoundly disrupted in the aging environment [200].

Furthermore, aging can alter the expression of chemokine receptors on axons, Schwann cells, and immune cells, rendering them less responsive to guidance cues [201, 202]. A seminal study by Zhou *et al.* establishes a compelling paradigm for how age-related chemokine changes can actively inhibit regeneration [163]. Their work demonstrates that chemokine ligand 13 (CXCL13) is the most markedly upregulated chemokine in aged mice following sciatic nerve injury. This upregulation depends on lymphotoxin-dependent phosphorylation of nuclear factor κB (NF-κB) [163]. CXCL13 attracts cluster of differentiation 8 (CD8+) T cells expressing its receptor 5 (CXCR5) to the vicinity of neurons that act as antigen-presenting cells by overexpressing major histocompatibility complex class I (MHC I) [203] (Fig. 3). Regeneration failure resulted from caspase3 activation induced by CXCR5+CD8+ T cells bound to MHC I-expressing sensory neurons. Notably, CXCR5+CD8+ T cells are increased in aged mice under physiological conditions compared to young mice [163, 204]. Reversing this change before injury or blocking signals such as CXCL13 after injury significantly improves axonal regeneration in aging organisms [163, 205]. This study illustrates that aging not only impairs beneficial chemokine signals but also actively induces maladaptive chemokine pathways that directly antagonize regeneration. Consequently, targeting these pathways—for example, by neutralizing CXCL13 after injury, blocking the recruitment of CXCR5+CD8+ T cells, or inhibiting caspase3 activation—represents a promising therapeutic strategy for reversing the decline in axonal regeneration in aging neurons [206, 207].

Therefore, the aged chemokine landscape is not merely passive but can actively orchestrate a hostile microenvironment that directly inhibits axon growth, shifting from impaired guidance to active repression of regeneration.

### Neuromuscular Junction in Neuronal Regeneration

The restoration of nerve function following peripheral nerve repair is contingent upon not solely axonal regeneration, but also the re-establishment of synaptic connections and neural circuits. Synaptic reconstruction and reinnervation subsequent to PNI present particularly formidable challenges [208], potentially resulting in a heightened rate of regeneration failure [209–211]. As a result, the clinical manifestations of PNI encompass aberrant sensory, motor, reflex, and autonomic functions in the innervation territory of the injured nerve [212].

The neuromuscular junction (NMJ) is particularly vulnerable to aging effects [213]. In uninjured peripheral neurons of aging organisms, synaptic protein expression is diminished. Numerous studies have demonstrated that aging induces significant NMJ degeneration and functional decline [214–217]. The molecular mechanisms underlying NMJ degeneration in aging have been extensively investigated. The upregulation of 15-hydroxyprostaglandin dehydrogenase (15-PGDH), a marker of aged tissue, has been identified in recent studies as an inhibitor of NMJ repair in nerves injured in aging [209, 218]. Specifically, in aged neurons, suppressed activity of the autophagy regulator mTOR disrupts autophagy, alters denervated muscle homeostasis, and hinders synaptic remodeling at the NMJ after injury [219, 220]. Beyond the intrinsic aging changes of the NMJ and the diminished survival rate of aging motor neurons [221], terminal SCs (tSC) are required for NMJ reinnervation following PNI [222–224]. However, as previously discussed, the reprogramming capacity of SCs can be affected by age, exacerbating the challenges in repairing aging nerves. Beyond these cell-specific challenges, the generalized bioenergetic insufficiency prevalent in the aged nervous system (as detailed in Section “Mitochondrial Dysfunction: The Energetic and Signaling Core of Impaired PNI Response in Aging”) presents an additional, systemic barrier to successful synaptic reconstruction and reinnervation at the NMJ. Macrophages, which play a constructive role in NMJ repair, also exhibit age-related functional decline, potentially contributing to failed NMJ reinnervation [225]. A growing body of research is currently exploring therapeutic strategies aimed at enhancing NMJ function [226, 227], including implanting bio-printed human skeletal muscle structures [227], which can be implemented in concert with other axonal regeneration therapies. Collectively, these emerging strategies highlight the necessity of combinatorial approaches that not only promote axon outgrowth but also specifically target the aged NMJ to achieve functional recovery.

In addition to motor nerve reinnervation, NP due to sensory nerve regeneration failure is also highly associated with age and injury [228–230]. For instance, TRPV1 channel-mediated pain in neuronal inflammation or injury is a burgeoning area of research interest [231, 232]. However, the extent to which aging intensifies sensory dysfunction following injury remains unclear, and the molecular mechanisms warrant further investigation [233]. PNI also causes clinical symptoms such as reflex changes and autonomic deficits [208, 234–236], but their aging-related changes have likewise not been studied in depth. Beyond the aforementioned regulatory mechanisms, epigenetic integration within the nucleus also plays important regulatory roles during axonal regeneration [4]. Even though this area is still in its infancy and its implications for aging in PNI are sparsely documented, it is evident that the aging peripheral nerve's regulatory response to injury is intertwined with these epigenetic processes [237]. In CNS studies, a related regulatory mechanism has been described, wherein reprogramming transcription factors can reverse the aging-associated changes in neurons by modulating DNA methylation patterns [238, 239]. In addition, studies on DRG have demonstrated that the regenerative responses vary among neuronal subtypes [240]. Consequently, understanding how the regenerative responses of different subtypes change with age is worthy of in-depth study.

## Mitochondrial Dysfunction: The Energetic and Signaling Core of Impaired PNI Response in Aging

The regenerative failure described earlier is largely attributable to bioenergetic insufficiency [241]. Mitochondria are not only the cellular power plants but also central hubs for calcium buffering and redox signaling [242]. The functional deterioration of mitochondria is a prominent early indicator of aging [243–245]. Their functional decline with aging, therefore, directly cripples the high-energy demands and finely tuned signaling required for successful axon regeneration. Interventions aimed at improving mitochondrial health in aging cells have shown potential for reversing various aging-related functional deficits [246]. Therefore, to understand how neuronal response pathways change with age, it is imperative to investigate how mitochondria themselves—and the myriad functions they perform during injury responses—are affected by aging [247, 248].

### Mitochondrial Dysfunction in Aging Neurons

**Fig. 4:**
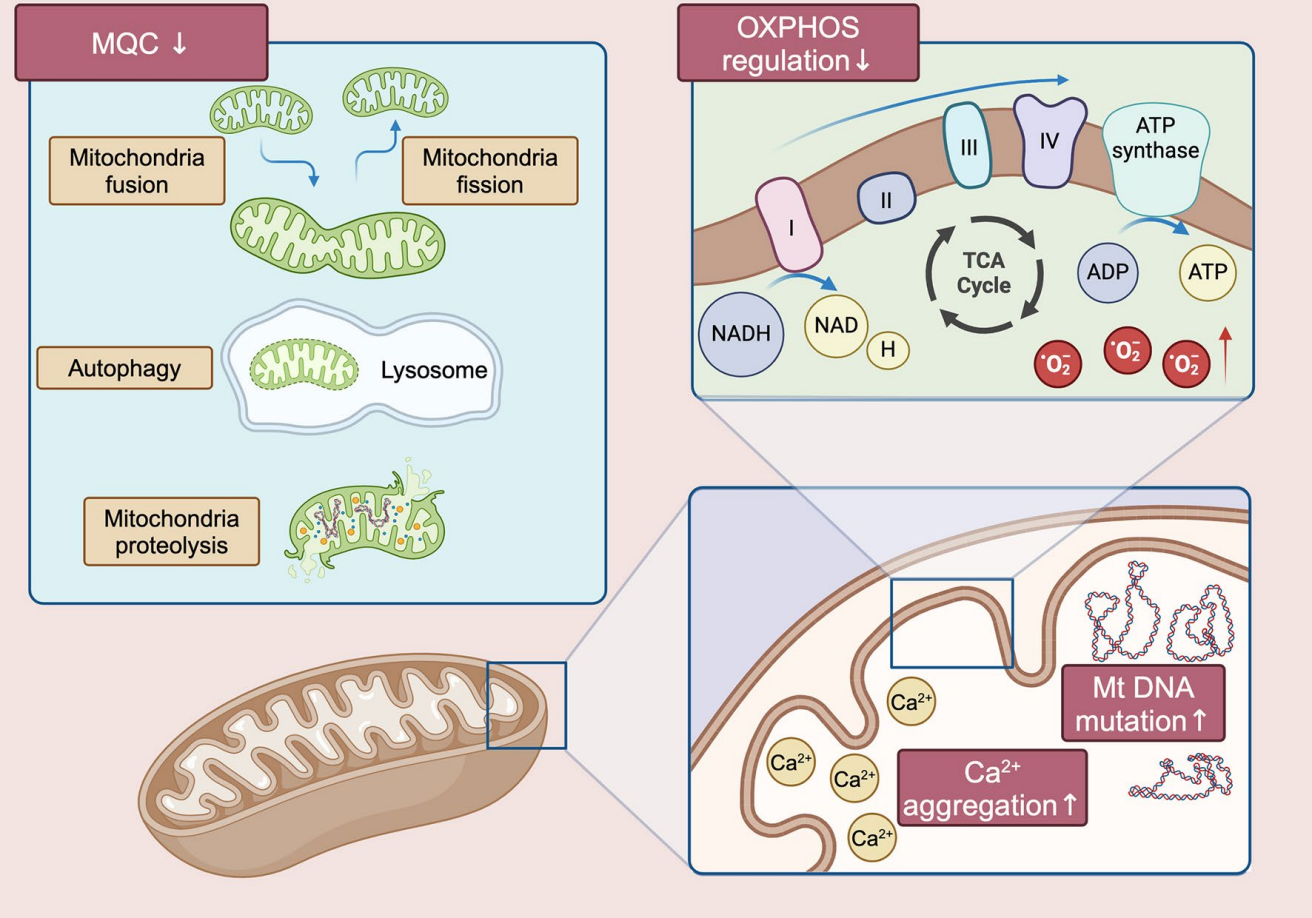
Multiple impacts of aging on mitochondria. Aging results in a perturbation of MQC, manifesting as physiological disruptions such as calcium overload, accelerated mtDNA mutation rates, and impaired OXPHOS. These alterations can significantly influence peripheral neuronal reactions to injury, attributed to the aberrant energy metabolism and the exacerbated oxidative stress. The blue arrows outside the text box represent down-regulated mitochondrial biological processes in the aged environment, and the red arrows indicate an increase in ROS.

Aging profoundly impairs mitochondrial quality control (MQC). This encompasses impaired mitochondrial fusion and fission. Additionally, aging reduces the autophagic clearance of dysfunctional mitochondria and diminishes mitochondrial respiratory capacity [249–252]. These disruptions severely affect a range of their biological functions. A plethora of research has illuminated the molecular underpinnings of these aging-induced alterations [36, 77, 243, 244, 253, 254], including an elevated rate of mitochondrial DNA (mtDNA) mutations [245], mitochondrial Ca^2+^ overload [77], an imbalanced nicotinamide adenine dinucleotide (NAD+)/nicotinamide adenine dinucleotide, reduced form (NADH) ratio [77, 244], and disruption of other mitochondrial homeostasis mechanisms [78] (Fig 4). Of interest, the aging-associated multifunctional signal mTOR, which plays roles in axon regeneration, inflammation mediation, and pain induction, also regulates mitochondrial function [82, 255–257]. Although mTOR is implicated in both aging and mitochondrial regulation, its precise role in repairing aged PNS remains unclear. Determining whether mTOR inhibition or modulation can improve mitochondrial health and repair outcomes in aged PNI models is a critical future direction [258].

Beyond their role in injury response, mitochondria must be efficiently transported along the axonal cytoskeleton to meet the high energy demands of distal regions [259, 260]. This mitochondrial transport is especially vital during axonal degeneration and regeneration subsequent to PNI [261]. This dynamic process is affected by aging. Aging-related changes in axonal transport are described in more detail in the following section.

### Mitochondrial Dysfunction Disrupts Neuronal Responses after Injury

As previously discussed regarding aging markers, the other nine hallmarks of aging are all associated with metabolic deficits [24]. Mitochondria are pivotal in orchestrating the neuronal injury response. ATP generation underpins processes including calcium signaling, autophagy, Wallerian Degeneration, retrograde transport of regeneration-associated factors, and pathways involved in transcription, translation, and energy metabolism [244, 262]. Mitochondrial dysfunction, a common consequence of aging, frequently culminates in a compromised cellular response.

Beyond mitochondrial dysfunction itself, substantial aging-associated ROS production also disrupts PNI responses [263]. The mitochondrial free radical theory of aging (MFRTA) emphasizes that ROS are instrumental in mitochondrial damage in aging cells and as a result of their dysfunction [77, 244, 264]. With advancing age, the stability of the mitochondrial respiratory chain complexes deteriorates, leading to reduced membrane potential, increased proton leakage, and ROS overproduction [265, 266]. The diminished activity of ROS-scavenging enzymes in aging cells further intensifies this challenge [267]. Oxidative stress, while potentially beneficial for axonal regeneration [268], can induce cellular dysregulation when excessive, affecting pathways such as MAPK-ERK, DLK-JNK, and STAT3 [181, 269]. This aging-induced phenomenon manifests as alterations in neuronal morphology, exacerbated dysregulation of intracellular calcium homeostasis, impaired synthesis and secretion of signaling proteins, which in turn regulate neuronal autophagy and other post-injury processes [37, 266]. It also directly leads to DNA damage [266, 270, 271], thereby accelerating a vicious cycle of cellular aging and mitochondrial dysfunction [272]. Notably, the dual role of ROS as both a necessary signaling molecule and a destructive agent creates a complex therapeutic landscape. The key challenge in the aged PNS is to define the precise thresholds that separate beneficial from detrimental ROS levels and to develop strategies to fine-tune this balance, rather than blanket inhibition [273, 274].

Beyond the deleterious signaling of ROS, the multifaceted mitochondrial deficits in aging neurons converge on a more fundamental failure: a severe bioenergetic crisis. Successful axon regeneration itself is an energetically demanding process requiring substantial ATP to fuel cytoskeletal remodeling, organelle transport, and synaptic reconstruction [275, 276]. However, the age-related impairments in oxidative phosphorylation (OXPHOS), reduced NAD+ levels, and a disrupted TCA cycle collectively cripple ATP production and deprive the cell of essential biosynthetic precursors[247, 277–279]. This energy failure acts as a core bottleneck, directly undermining the high-energy demands of the repair processes discussed throughout this review, from calcium clearance to autophagy and axonal transport.

A growing number of studies report that interventions targeting the previously mentioned pathways, such as supplementation of NAD+ [280], enhancement of MQC [281], reversal of mitochondrial autophagy inhibition [282–284], and suppression of oxidative stress [285], significantly contribute to reducing mitochondrial damage or rectifying the impaired neuronal response functions after injury in aging models. Although most evidence comes from CNS or in vitro studies, and direct evidence in PNS injury models is limited, reversing mitochondrial dysfunction to treat injury response deficits in aging neurons remains a significant direction for future research [283, 286, 287]. Key unresolved questions include: What is the role of mitochondrial endoplasmic reticulum contact site (MERCS) dysfunction in the age-related exacerbation of calcium dysregulation after axonal injury [288, 289]? Does modulating MERCS integrity offer a novel therapeutic strategy [290]? Are mTOR-driven mitochondrial defects reversible in vivo [291]? Resolving these issues will be essential for developing targeted interventions to restore mitochondrial health and function in the aged PNS.

## Aging-related Disruption of Neuronal Transport Systems: Compromising Communication in PNI

The efficient transport of injury signals, organelles, and building materials is the lifeline of the PNI response. Axonal transport undergoes adaptive changes following nerve injury, which are indispensable for the orchestrated neuronal degeneration and the subsequent axonal regeneration [292, 293]. This process relies on the integrity and function of the neuronal cytoskeleton as well as the action of motor proteins [294]. Age-related deterioration of the cytoskeleton and motor proteins disrupts this vital communication, explaining the impaired retrograde signaling and inadequate delivery of regenerative machinery to the growth cone mentioned in Sections “Aging Affected Neuronal Regenerative Processes” and “Mitochondrial Dysfunction: The Energetic and Signaling Core of Impaired PNI Response in Aging” [43, 137].

### Changes in the Peripheral Neuronal Cytoskeleton

**Fig. 5:**
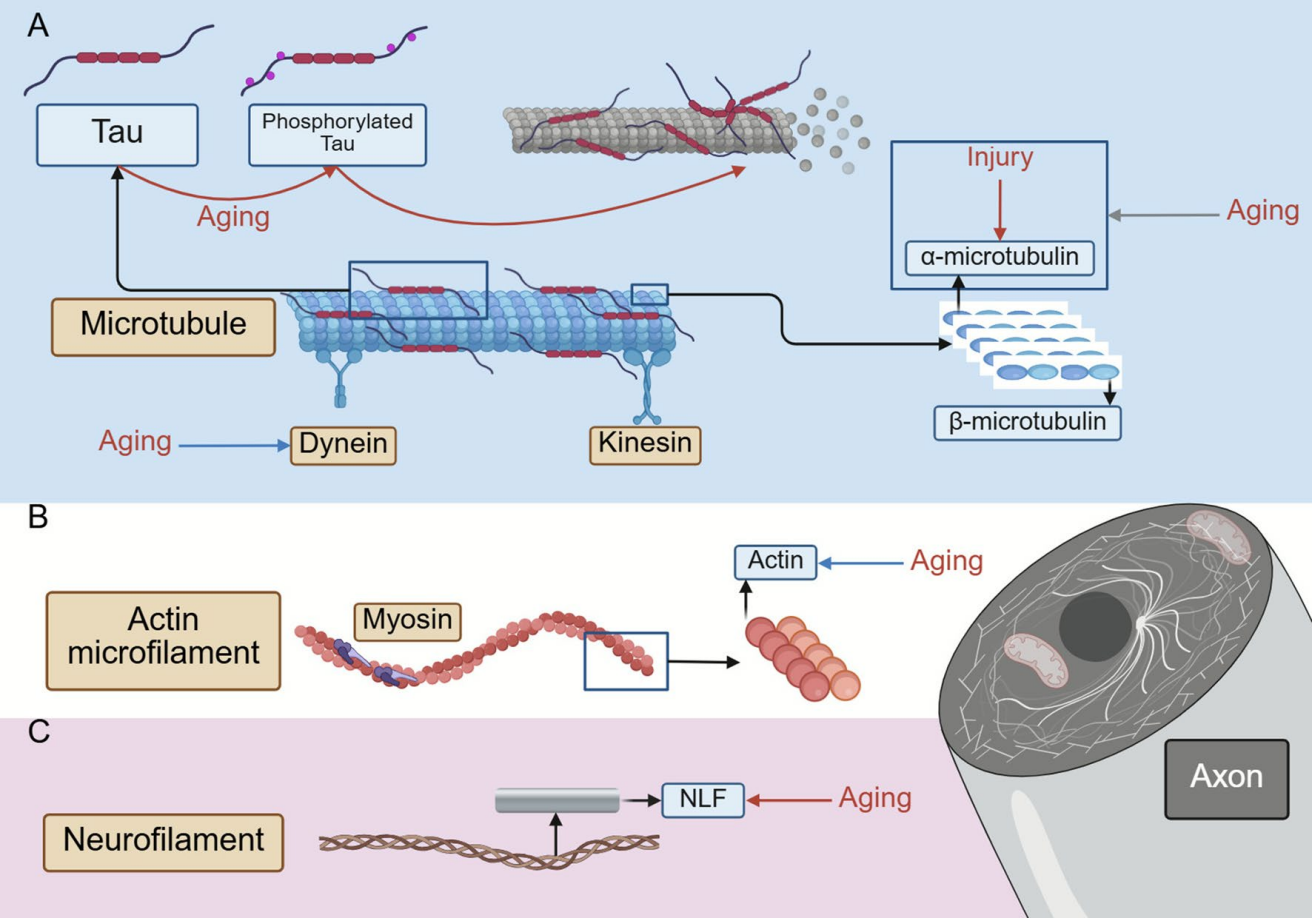
Changes in aging neurons by a transport system that plays an important function in neuronal repair. The neuronal skeleton includes microtubules, actin microfilaments, and neurofilaments, as well as associated motor proteins. Their aging-dependent changes are the classical manifestation of neurological aging. Aging-dependent changes in the neuronal transport system are highly dependent on degenerative changes in the neuronal skeleton and other multifaceted influences, such as changes in mitochondrial energy supply. Red arrows represent facilitation of a biological process or substance by the corresponding factor, while blue arrows represent inhibition. Gray arrows represent the key impact trend to be elucidated.

The neuronal cytoskeleton, composed of microtubules, actin microfilaments, and neurofilaments, is essential for maintaining neuronal integrity and facilitating intracellular transport. Significant aging-related changes occur in these components, impacting their stability and function [217, 295–297] (Fig. 5).

Microtubule dynamics are crucial for initiating retrograde signaling after injury [298]. This process is largely dependent on microtubule structure, function, and plasticity, all of which are attenuated in aging neurons. Previous experimental evidence suggests that both the abundance and stability of microtubule proteins decrease with age [217]. This decline may be attributed to the significant age-dependent reduction and hyperphosphorylation of Tau proteins, which are crucial for microtubule stability [217, 299]. Evidence points to the elevated BMP pathway activity and the suppression of PI3K/AKT signaling by Nr4a1 as central initiating events, leading to tau hyperphosphorylation [300, 301]. This is exacerbated by a kinase-phosphatase imbalance favoring hyperphosphorylation, inflammatory calcium dysregulation that promotes early p-tau accumulation, and a decline in the protective O-GlcNAc modification of tau protein [302, 303]. Numerous studies have attempted to reverse aging-related neuronal dysfunction by targeting Tau to improve microtubule dynamics but the therapeutic benefits of such interventions remain limited [304]. Of interest, injury promotes retrograde signaling by increasing tyrosine α-microtubulins [305], and the relevance of this change in aging warrants further investigation (Fig. 5A).

Aging also attenuates the role of actin microfilaments in axoplasmic transport dynamics by down-regulating the expression of actin, a major component of actin microfilaments, and disrupting its structural integrity [296, 306]. Compared to 6-month-old rats, 24-month-old rats show a 37 % decrease in actin expression [307]. The regulation of motor protein gene expression involves multiple aging-related pathways [308]. Research indicates that F-actin accumulates in the fruit fly brain with age, and lifespan-extending interventions can reverse this phenomenon [309]. In the spinal cord of aged dogs, increased expression of inflammatory genes, along with altered microglial polarization, may indirectly impact transport functions in motor neurons [310]. However, PNS-specific experimental data are still lacking. Aging-related actin is hypothesized to form structurally unstable filaments, impairing its function in neuronal transport [296]. Additionally, modulating actin dynamics has emerged as a promising drug target for counteracting neuronal aging and potentially extending neuronal lifespan [311, 312] (Fig. 5B). Critically, emerging evidence suggests that pharmacological interventions aimed at stabilizing actin filaments or promoting their dynamics (e.g., through targeting regulators like cofilin or profilin) can enhance axonal transport and growth cone motility [309, 313, 314]. Whether these strategies improve functional recovery, such as accelerated reinnervation and sensory-motor restoration, in aged PNI models remains a compelling and clinically relevant area for future investigation.

Neurofilament homeostasis is another important prerequisite for efficient axonal transport [315–317]. The exponential aging-related increase in neurofilament light chain (NFL), a critical subunit of neurofilament proteins, may suggest an accelerated neurofilament turnover [318–320] (Fig. 5C). It remains an open question whether this increase in NFL is a primary driver of transport deficits—for instance, by disrupting the cytoskeletal network and impeding the movement of motor proteins—or a secondary, compensatory response to age-related axonal stress [321, 322]. Some studies posit that increased neurofilament degradation might be an attempt to clear damaged components and maintain axonal caliber, albeit insufficient to prevent overall functional decline [323, 324].

### Changes in Motor Proteins

Motor proteins, including dynein, kinesin, and myosin, are crucial for mediating the transport of cellular cargo along the cytoskeleton. Dynein and kinesin function in conjunction with microtubules, while myosin operates along actin microfilaments (Fig. 5).

The retrograde transport of injury signals from the injury site to the neuronal cytosol in response to mitochondria and dynein is known as retrograde axoplasmic transport, which is critical in the neuronal injury responses [260, 325, 326]. Factors, including c-Jun, ATF3, Smad, STAT3, ERK, and XBP1, are required for retrograde transport in the presence of the neuronal cytoskeleton and actin. Dynein function is energy-dependent and thus compromised by the age-related mitochondrial dysfunction described previously [327, 328]. Aging also disrupts intra-axonal transport by impairing dynamin-actin interactions, leading to dynamin dysfunction [329–331], although the underlying molecular mechanisms remain to be fully elucidated. Additionally, aging of dynamin can further exacerbate mitochondrial dysfunction [332].

The efficacy of kinesin and myosin in facilitating anterograde axoplasmic transport is also compromised by aging [331, 333]. This decline impacts the transport of signals integral to epigenetic regulation, which is itself inhibited with aging [4]. Reduced motor protein activity can be largely ascribed to generalized aging-related alterations in protein properties and cytoskeletal coordination [217]. Furthermore, aging may impact the transcriptional regulation of motor protein genes [334]. For instance, in skeletal muscle aging, ATF4 (a transcription regulatory protein) has been shown to rapidly induce muscle atrophy in young animals, suggesting it may participate in age-related muscle wasting by regulating the expression of myosin-related genes [335]. However, the underlying mechanisms in the PNS remain unclear.

## Aging-induced Hyperinflammation: Corrupting the Microenvironment for PNI Repair

A controlled inflammatory response is essential for clearing debris and initiating repair after PNI [336]. However, aging promotes a shift toward chronic, dysregulated inflammation. Biopsies of aged peripheral nerves reveal demyelination, axonal degeneration, and heightened inflammation accompanied by tissue fragmentation [337]. Even in the uninjured state, the aging neural microenvironment exhibits elevated pro-inflammatory markers, increased cytokine release, and macrophage infiltration [138]. Additionally, SASP, a well-documented driver of sterile inflammation in aged tissues, is readily detected [338]. This leads neurons to adopt a hyperinflammatory phenotype characterized by sustained cytokine production and increased ROS generation [339]. This state is influenced by various factors, including altered ion channel activity, increased reactive oxygen species generation, abnormal chemokine signaling, and mitochondrial dysfunction [340].

This persistent hyperinflammation state interferes with the full course of PNI response [134]. It impairs normal neuronal function and structural integrity [341], leading to pathological protein aggregation [342], neuronal degeneration [343], autophagy dysfunction [344], and even apoptosis [345]. Within the PNI response signaling pathway, this hostile microenvironment actively suppresses the reparative functions of SCs and macrophages (Section “Dysregulation of Wallerian Degeneration”) and directly exposes regenerating axons to elevated levels of inhibitory cytokines, thereby obstructing the regenerative processes outlined in Section “Aging Affected Neuronal Regenerative Processes” [346]. Consequently, the dysregulation of inflammation has profound implications for the neuronal response to injury. Notably, the suppression of inflammation in aging neurons has been found to ameliorate cellular structure and function [337], such as oral administration of colony-stimulating factor 1 receptor (CSF1R) [347]. This underscores the potential of anti-inflammatory therapies as a promising avenue for enhancing the reparative capacity of aging neurons. Furthermore, it is important to note that the maintenance of an appropriate inflammatory environment also underlies axonal regeneration after PNI [348, 349]. For example, Wallerian Degeneration is inherently linked to the inflammatory milieu and its associated factors [350]. In spinal cord injury models, IL-6 supports functional neurological recovery by promoting axonal sprouting [351]; IL-4, a type 2 cytokine, significantly inhibits the senescence process in macrophages [352]. However, the balance between its pathological inflammatory and neuroprotective effects requires careful regulation. Balancing this inflammatory equilibrium during clinical interventions is a critical challenge and a focus in biomaterials research [353, 354].

The adaptive immune gene profiles also undergo extensive changes after injury to aging neurons. A recent study found that differentially expressed genes post-injury were strongly enriched in immune response pathways, with age serving as a critical variable [163]. From an immune cell perspective, compared to young rats, aging rats after sciatic nerve injury show a rapid increase in neutrophil proportions rather than monocytes, alongside a significantly delayed increase in M2 macrophages [119]. These alterations subsequently affect Wallerian Degeneration and axonal regeneration. Research on immune responses in neuronal injury is an evolving field requiring deeper mechanistic exploration.

## Aging-Related Changes in Other Non-neuronal Factors in the Microenvironment

Microenvironmental factors, encompassing biochemical, spatial, and biomechanical elements, exert a significant regulatory influence on PNI response. This microenvironment is composed of the extracellular matrix, various cells, including SCs and macrophages, and diverse soluble signaling molecules [355]. Extensive research has been dedicated to understanding neuron-microenvironment interactions, revealing dynamic cellular interplay and underlying mechanisms in PNI response [356]. Several aging-related potential factors at the injury site lead to response deficits, such as axonal degeneration and regeneration dysregulation, including extracellular matrix sclerosis and degradation [357, 358], and changes in exocytosis [359].

Beyond the Büngner band, long recognized as the optimal regenerative microenvironment after PNI [360], recent studies have increasingly highlighted the significant roles of satellite glia cells (SGCs), vascular endothelial cells, and fibroblasts in modulating the injury response cascade.

SGCs are integral constituents of the PNS, with previous research primarily focusing on their involvement in peripheral NP [361]. Their molecular contributions to peripheral nerve regeneration have not been extensively explored, primarily due to a lack of specific biomarkers. However, emerging studies indicate that SGCs facilitate regeneration, partly through activating peroxisome proliferator-activated receptor α (PPARα) signaling [362]. PPARα is closely associated with organismal aging and cellular senescence mechanisms. It represents a potential pharmacological target for reversing the biological manifestations of cellular aging [363]. Nonetheless, the specific molecular regulatory mechanisms of SGCs in peripheral nerve repair and their aging-related changes remain poorly elucidated, presenting a key future research direction [10, 364].

Endothelial cells foster a nerve-reparative microenvironment, primarily by forming intra-neural blood vessels [356]. Exosomes, as important intercellular messengers in the nervous system, are believed to significantly influence the regulation of the PNI response [365]. Recent research highlights endothelial cell-derived exosomes (EC-EXOs), which activate the PI3K signaling pathway to promote pro-repair phenotypes in SCs. EC-EXOs promote this more effectively than SC-derived exosomes [162], which have been implicated in neuronal injury through pathways such as the STAT3 [366]. Collectively, EC-EXOs may promote functional recovery following PNI by inhibiting neuronal apoptosis and fostering axonal repair, myelin regeneration, and angiogenesis. However, the exocrine function of aging endothelial cells can be significantly impaired and is even associated with the development of aging-related diseases [367]. In addition, exosomes from various origins are a prominent research topic in the field of neural repair, with a discernible link to the aging process. They mediate age-related cellular changes, and their properties (e.g., quantity, cargo) are altered by cellular aging [359, 366]. Their therapeutic potential lies in counteracting neuronal aging [359].

Fibroblasts derived from the PNS are integral to the structure of the inner and outer nerve sheaths and the perineurial membranes. They play a pivotal role in the growth and maturation of motor neurons by secreting neurotrophic factors [368, 369]. Fibroblast growth factors, in particular, show promise in natural nerve conduit therapy [370]. Skin fibroblast aging is known to induce dysfunction in other skin cells and may trigger systemic inflammation [371]. Similarly, cardiac fibroblasts age, becoming activated and contributing to fibrosis and dysfunction [372]. Follow-up studies could delve deeper to explore whether PNS fibroblasts undergo similar changes, potentially impairing repair after PNI.

A considerable body of research has focused on modulating the microenvironment to facilitate axon repair following PNI. Strategies such as the use of aligned endothelium [373] and exosomal therapies [374] have been employed to address peripheral nerve repair deficiencies. For instance, the transplantation of platelet-rich plasma-derived exosomes treated MSCs (pExo-MSCs) markedly enhances axonal regeneration, remyelination, and the functional recovery following PNI [375]. Other studies explore enhancing nerve regeneration by manipulating the electrical and immune microenvironments [355, 376]. For example, bioelectrically active materials significantly promote repair and restore function, most attributed to the promotion of macrophage polarization from the M1 to the M2 phenotype [377, 378].

## Conclusion

This review has synthesized the multifaceted impact of aging on the peripheral nervous system's response to injury. A central theme that emerges is that aging does not simply weaken the individual processes of degeneration and regeneration but fundamentally corrupts the intricate signaling network that orchestrates them. We have delineated how age-related dysregulation of pivotal early signals, such as Ca^2+^, initiates a cascade of failure, compromising autophagy and Wallerian Degeneration. Subsequently, the regenerative phase is thwarted by impaired retrograde transport, a diminished transcriptional response, and a hostile environment.

The overarching implication of these findings is that the regenerative failure in aging is a systems-level disorder. It arises from the synergistic dysfunction of neurons, Schwann cells, and immune cells, all operating within a compromised microenvironment characterized by mitochondrial insufficiency, hyperinflammation, and deficient trophic support. This integrated perspective reveals that therapeutic strategies aiming to reverse a single deficit are likely to be insufficient. Instead, combinatorial approaches that simultaneously target multiple hallmarks of aging—such as improving mitochondrial health while dampening maladaptive inflammation—hold the greatest promise for restoring repair competence.

In conclusion, advancing our understanding of the aged PNI response necessitates a shift from studying isolated mechanisms to investigating their interactions. Overcoming the challenge of nerve repair in the elderly will depend on our ability to reconstitute a youthful, coordinated molecular and cellular dialogue within the injured nerve.

## Limitations and Perspective

The relationship between aging and the diverse signaling pathways involved in the injury response remains insufficiently elucidated. The intricate interplay among diverse regulatory pathways offers a rich field for scientific inquiry. Epigenetic mechanisms exert a significant influence over neural regeneration, yet the impact of aging on the epigenetic regulation of DNA methylation, histone modifications, and noncoding RNA following PNI remains largely uncharted territory.

In addition to delving deeper into the regulatory pathways whose changes are not yet fully understood, a significant trend in forthcoming research will involve identifying intervention targets and developing strategies to artificially counteract aging-associated alterations. Axonal repair and clinical healing after PNI are important not only for the recovery of motor and sensory functions, but also for mitigating the formation of neuromas and the abnormal sensations they cause [379]. The pronounced aging-related differences in responses to PNI necessitate a more challenging approach to injury repair in the elderly. Studies suggest the need for targeted interventions tailored to neural repair processes affected by aging [163]. For example, the advancement of biomaterials such as neural guide conduits (NGCs) could be enhanced by incorporating biological factors designed to counteract the detrimental effects of aging [380, 381].

The multidimensional impact of aging on organisms and the repair bio-behavior of peripheral neurons are both significant domains of research in the life sciences. A comprehensive examination of the nexus between these factors and the exploration of biological therapeutic approaches is a profound subject for future investigation.
